# Safety, Pharmacokinetics, and Efficacy Study of Porcine and Rabbit Antithymocyte Globulin in the Treatment of Patients With Severe Aplastic Anemia: A Multicenter, Prospective, Randomized, Open‐Label Clinical Trial

**DOI:** 10.1002/mco2.71084

**Published:** 2026-10-17

**Authors:** Yawen Zhang, Yingying Zhang, Yuemin Gong, Yu Sun, Guangsheng He

**Affiliations:** ^1^ Department of Hematology Jiangsu Province Engineering Research Center of Cell and Gene Therapy for Hematologic and Lymphoid Diseases The First Affiliated Hospital with Nanjing Medical University, Jiangsu Province Hospital Nanjing China; ^2^ Department of Hematology The Second Affiliated Hospital of Soochow University Suzhou China

**Keywords:** clinical trial, immunosuppressive therapy, porcine antithymocyte globulin, rabbit antithymocyte globulin, severe aplastic anemia

## Abstract

Severe aplastic anemia (SAA) is a T lymphocyte‐mediated bone marrow failure disorder. Only porcine ATG (p‐ATG) and rabbit ATG (r‐ATG) are available in mainland China, with the optimal first‐line regimen undetermined. This study compared their pharmacokinetics, efficacy, and safety. Ninety‐three SAA patients ineligible for transplantation or lacking a suitable donor (p‐ATG, *n* = 55; r‐ATG, *n* = 38) who were receiving immunosuppressive therapy were prospectively enrolled between 2020 and 2024. The primary endpoint was dynamic changes in lymphocyte subsets. Both ATGs peaked on treatment Day 5; r‐ATG cleared significantly faster, with comparable peak active concentrations. The p‐ATG group showed a stable early neutrophil increase, whereas the r‐ATG group had an initial decline, with comparable recovery at 1 month. The r‐ATG group exhibited prolonger lymphopenia, lower CD3^+^/CD4^+^ T cells, reduced Treg cells, and higher CD19^+^ B‐cell proportions. IL‐10 was significantly elevated on day 2 after ATG administration. At 6 months, the CRR was 18.87% with p‐ATG and 18.92% with r‐ATG; ORR was 69.81% with p‐ATG and 56.76% with r‐ATG. Two‐year ORR and overall survival were similar, with no significant differences in infection or serum sickness. P‐ATG and r‐ATG have distinct pharmacokinetic and immunomodulatory but comparable efficacy and safety profiles, supporting interchangeable use in clinical practice.

## Graphical abstract

This prospective trial compared porcine‐ATG and rabbit‐ATG for severe aplastic anemia. Both agents peaked on Day 5, with faster clearance for rabbit‐ATG. Porcine‐ATG induced an early stable neutrophil rise, whereas rabbit‐ATG caused an initial neutrophil drop. The two groups showed distinct immune‐suppression patterns, yet achieved comparable 6‑month response, 2‐year survival, infection, and serum‐sickness rates. These two ATG products are interchangeable clinically.

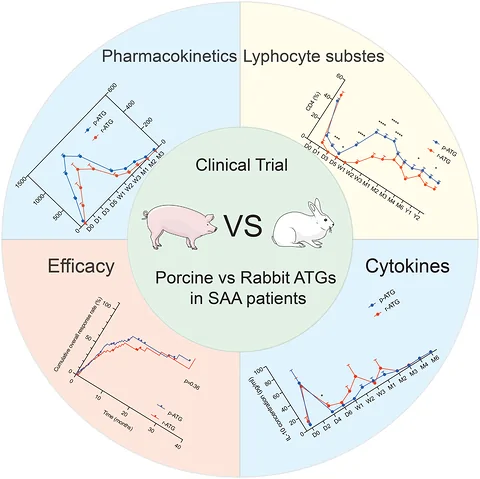

## 1 Introduction

Aplastic anemia (AA) is a bone marrow failure disorder characterized by pancytopenia, and patients are stratified into non‐severe AA (NSAA) and severe AA (SAA) according to peripheral blood cell counts [1, 2]. The incidence of acquired aplastic anemia is approximately 2 per million annually in Europe and North America, with rates two to three times higher in East Asia [2]. It is widely accepted that T‐lymphocyte‐mediated severe depletion of hematopoietic stem cells constitutes the primary cause of this disease. Activated autoreactive T cells target hematopoietic stem and progenitor cells and are accompanied by excessive secretion of myelosuppressive inflammatory cytokines. Dysfunctional regulatory T cell (Treg) activity exacerbates autoimmune‐mediated bone marrow injury, ultimately leading to severe bone marrow hypoplasia and hematopoietic failure [3, 4].

According to the latest guidelines, first‑line standard therapy for adult patients with newly diagnosed acquired SAA consists of antithymocyte globulin (ATG)‑based immunosuppressive therapy (IST) combined with eltrombopag in the absence of an HLA‑matched sibling donor [2, 5, 6, 7]. In mainland China, herombopag has also been approved for the treatment of patients with SAA in combination with IST [8]. Clinical studies on the efficacy and immunologic characteristics of various ATG formulations have been conducted. Rabbit ATG (r‑ATG) exerts a more potent inhibitory effect on lymphocytes than horse ATG (h‑ATG) and is associated with slower CD4^+^ T‑cell recovery, which may lead to inferior efficacy in patients with SAA [9, 10]. Accordingly, h‐ATG has been the most widely preferred ATG agent worldwide.

However, h‐ATG has been unavailable in mainland China, since 2000, and porcine ATG (p‐ATG) was developed in 1983 and approved for the treatment of AA in 2004. Currently, only porcine ATG (p‐ATG) and r‐ATG are available, and the optimal first‐line ATG formulation remains to be determined. Several retrospective studies have compared the efficacy of p‐ATG and r‐ATG, yielding somewhat inconsistent results: some investigations reported superior time‐dependent efficacy, as evidenced by a higher 3‐month overall response rate (ORR) in the p‐ATG group relative to the r‐ATG group, whereas other datasets indicated comparable therapeutic effects between the two agents [11, 12]. Therefore, a prospective comparative study is still required to determine the difference in efficacy between these two regimens.

Meanwhile, fundamental questions regarding these two ATG preparations remain poorly addressed. Few studies have systematically explored the dynamic discrepancies in in vivo lymphocyte subset depletion and immune reconstitution patterns after p‐ATG versus r‐ATG administration. Moreover, their distinct pharmacokinetic properties have not been quantitatively characterized in SAA populations, which restricts our understanding of their divergent immunomodulatory effects. Therefore, we conducted a prospective study to compare the pharmacokinetics, therapeutic efficacy, and dynamic changes in peripheral blood cell counts and cytokine levels in SAA patients treated with p‐ATG or r‐ATG in combination with cyclosporine A (CsA) and thrombopoietin receptor agonists (TPO‐RAs). This study provides evidence elucidating the in vivo effects of both ATG formulations and guiding their clinical application in the treatment of SAA.

## 2 Results

### 2.1 Demographic Characteristics of AA Patients

Between November 2020 and April 2024, 93 AA patients were enrolled and received intensive immunosuppressive therapy (IST). Among them, 55 patients received p‐ATG and 38 received r‐ATG. The trial participant screening, randomization, follow‐up, and exclusion process is summarized in the CONSORT flow diagram (Figure S1). Baseline characteristics of the two groups before treatment are shown in Table 1. Note that 13 patients in the p‐ATG group and 11 patients in the r‐ATG group had very severe aplastic anemia (VSAA). There were no significant differences in demographic or clinical characteristics between the two groups (Tables 1 and 2). We also used SMD calculations to quantify the magnitude of intergroup baseline imbalance across the two cohorts: Neutrophil and platelet counts were adequately balanced at baseline (SMD < 0.1). Mild intergroup imbalance was observed in white blood cell count, hemoglobin, and reticulocyte count (0.1 ≤ SMD ≤ 0.3). A noticeable baseline imbalance was detected in age and total lymphocytes (all SMDs > 0.3). The median follow‐up was 25.53 months (range, 0.23–56.90 months) for all patients.

**TABLE 1:** Baseline characteristics of SAA patients.

	p‐ATG (*n* = 55)	r‐ATG (*n* = 38)	Overall (*n* = 93)	*p* value
Age (median, IQR)	41.00 (20.00, 54.25)	48.50 (26.75, 61.00)	44.00 (23.00, 56.00)	0.12
Gender (male)	28 (50.90%)	20 (52.60%)	48 (51.61%)	0.87
Disease stage				
SAA	42 (76.36%)	27 (71.05%)	69 (74.19%)	0.57
VSAA	13 (23.64%)	11 (28.95%)	24 (25.81%)	0.57
TPO‐RA use				
Eltrombopag	36 (65.45%)	22 (57.89%)	58 (62.37%)	0.46
Herombopag	19 (34.55%)	16 (42.11%)	35 (37.63%)	0.46
Infection before ATG	20 (36.36%)	16 (42.11%)	36 (38.71%)	0.58
Infection after ATG within 1 month	22 (40.00%)	20 (52.63%)	42 (45.16%)	0.23
PNH clone	5 (9.09%)	4 (10.53%)	9 (9.68%)	0.82

Abbreviations: AA, Aplastic anemia; IST, intensive immunosuppressive therapy; NSAA, non‐severe AA; p‐ATG, porine‐antithymocyte globulin; r‐ATG, rabbit‐antithymocyte globulin; PNH, paroxysmal nocturnal hemoglobinuria; SAA, severe AA; TPO‐RA, thrombopoietin receptor agonist; VSAA, very severe AA.

**TABLE 2:** Peripheral blood cells and lymphocyte subsets in SAA patients.

	p‐ATG (*n* = 55)	r‐ATG (*n* = 38)	*p* value
ANC (×10^9^)	0.88 (0.28, 1.64)	0.65 (0.19, 1.58)	0.80
ALC (×10^9^)	1.45 (0.89, 2.16)	1.14 (0.61, 1.64)	0.05
Hb (g/L)	68.00 (63.00, 72.50)	69.00 (62.75, 73.50)	0.44
PLT (×10^9^)	21.00 (11.50, 32.00)	23.50 (12.75, 38.00)	0.82
RET (×10^9^)	19.50 (9.23, 41.90)	15.50 (5.75, 30.00)	0.20
CD3+ (%)	78.30 (70.74, 82.40)	78.70 (58.80, 84.00)	0.22
CD3^+^CD4^+^ (%)	42.40 (32.60, 50.80)	48.05 (39.40, 59.20)	0.13
CD3^+^CD8^+^ (%)	25.20 (18.80, 34.30)	26.95 (13.65, 34.85)	0.92
CD19^+^ (%)	11.90 (6.20, 19.95)	12.60 (8.20, 20.80)	0.15
CD4^+^CD25^+^CD127^−^ (%)	4.60 (3.05, 6.30)	4.30 (2.10, 5.70)	0.54

*Note*: All data were shown as median (IQR). All data were collected 1 day before ATG administration.

Abbreviations: ALC, absolute lymphocyte count; ANC, absolute neutrophil count; Hb, hemoglobin; PLT, platelets; RET, reticulocytes.

### 2.2 Post‐Administration Concentration Dynamics In Vivo

To evaluate the pharmacokinetics of ATG after IST, we measured serum ATG concentrations on Days 0, 1, 3, 5, 7, 14, 21, and 28, as well as at 2 and 3 months following treatment (Figure 1A). In both the p‐ATG and r‐ATG groups, serum ATG concentrations peaked on Day 5 (p‐ATG: 1248.84 µg/mL; r‐ATG: 343.93 µg/mL). Post‐peak, r‐ATG demonstrated significantly faster clearance: its concentration dropped by 99.80% 1 month after treatment, whereas p‐ATG showed a more moderate 94.36% reduction. To further quantify the active ATG, we used a methodology that integrates flow cytometry and ELISA, as described previously [13]. Similar to total ATG levels, active ATG concentrations peaked on Day 5 in both groups, and r‐ATG showed significantly faster clearance (Figure 1B). Notably, active ATG concentrations were comparable between the p‐ATG (34.42 ug/mL) and r‐ATG (33.26 ug/mL) groups, differing from total ATG concentrations.

**FIGURE 1:**
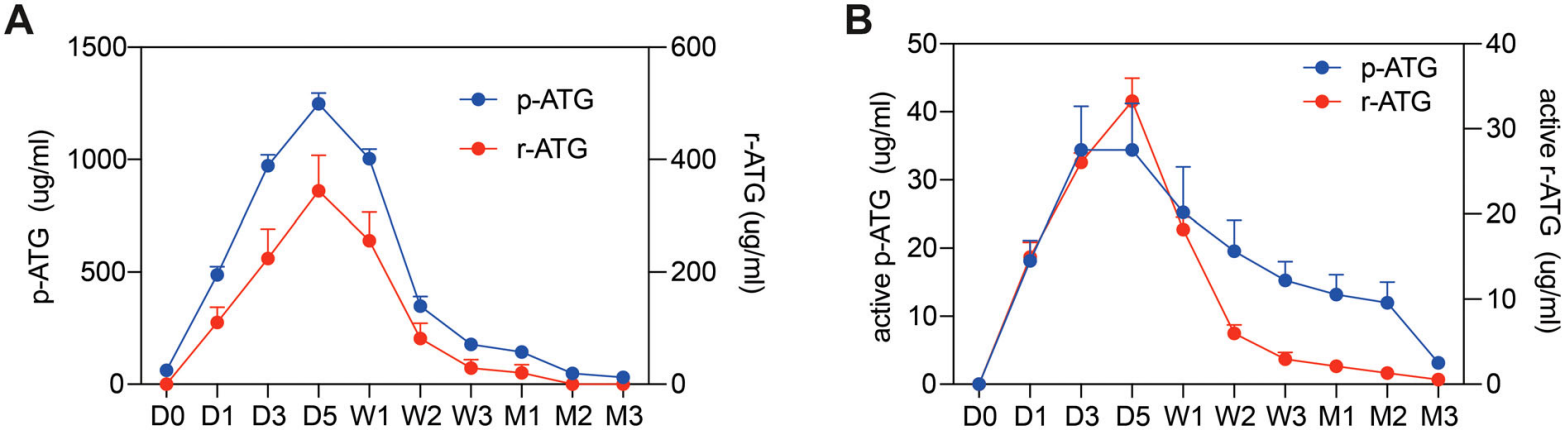
The pharmacokinetics of ATG after IST. (A) Serum p‐ATG and r‐ATG concentrations following treatment (p‐ATG, *n* = 13; r‐ATG, *n* = 10). (B) Active ATG concentrations detected by a methodology integrating flow cytometry and ELISA [13] (p‐ATG, *n* = 31; r‐ATG, *n* = 22).

### 2.3 In Vivo Long‐Term Dynamics of Neutrophil and Lymphocyte Subsets

We compared complete blood cell counts from baseline to 2 years in all patients treated with p‐ATG or r‐ATG. Administration of p‐ATG was associated with a stable increase in absolute neutrophil counts during the first week, which differed markedly from its effect on lymphocytes (Figure 2A). In contrast, r‐ATG reduced absolute neutrophil counts within the first 5 days after treatment, consistent with its effect on lymphocytes. One month after treatment, both p‐ATG and r‐ATG were associated with similar changes in absolute neutrophil counts (Figure 2A). Both p‐ATG and r‐ATG are polyclonal antibodies directed against a broad range of cell‐surface antigens and exhibit potent in vitro lymphocytotoxic activity. Both agents induced rapid lymphocyte depletion, although lymphopenia persisted significantly longer with r‐ATG, lasting up to 6 months after treatment (Figure 2B). Total lymphocyte counts were similar between the two groups from 1 year after IST onward. We subsequently analyzed lymphocyte subsets by flow cytometry. Compared with patients who received p‐ATG, those treated with r‐ATG had lower CD3^+^ and CD4^+^ T‐cell counts throughout nearly the entire 2‐month period (Figure 2C,D). This difference in CD4^+^ T‐cell counts persisted at 1 year after IST treatment. Smaller differences were observed in the kinetics of CD8^+^ T‐cell depletion and reconstitution between the two groups (Figure 2E). Regulatory T cells (defined as CD4^+^CD25^+^CD127^−^) were significantly lower within 1 month after r‐ATG therapy (Figure 2F). From the third week to several months after treatment, the percentage of B cells (CD19^+^) was higher in patients treated with r‐ATG than in those treated with p‐ATG (Figure 2G).

**FIGURE 2:**
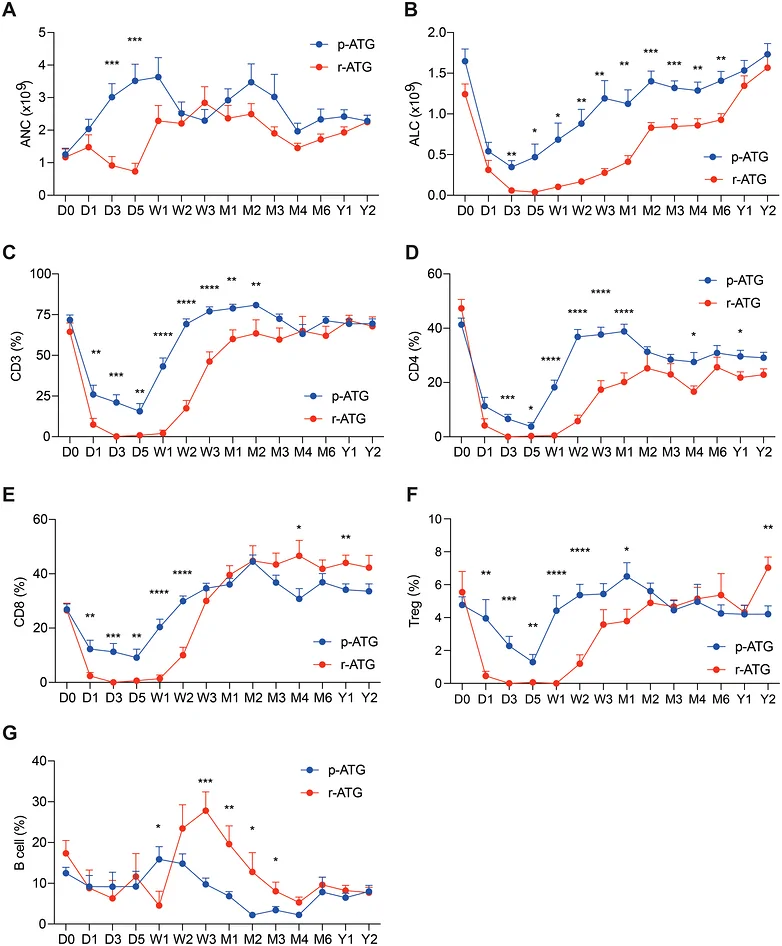
Dynamic changes in complete blood cell count from baseline to 2 years in all patients treated with p‐ATG or r‐ATG. (A–G) Changes in absolute neutrophil count, absolute lymphocyte count (ALC), percentages of CD3^+^, CD3^+^CD4^+^, CD3^+^CD8^+^, regulatory T cells (Tregs), and B cells (p‐ATG, *n* = 55; r‐ATG, *n* = 38).

### 2.4 Long‐Term Hematological Responses

We assessed the hematological responses at 1, 3, and 6 months and at 1 and 2 years after IST (Figure 3A–C). The ORRs were comparable between p‐ATG and r‐ATG at all time points within 2 years. The hematologic response rate was 69.81% with p‐ATG and 56.76% with r‐ATG at 6 months, and 73.17% with p‐ATG and 74.07% with r‐ATG at 2 years. At 3 months, the partial response rate (PRR) was higher in patients treated with p‐ATG than in those treated with r‐ATG (51.85% vs. 29.73%; *p* = 0.02), whereas the ORRs were comparable between the two groups. After adjustment for imbalanced baseline variables in multivariate logistic models, there were no significant differences in ORR between the p‑ATG and r‑ATG groups at 1, 3, 6, 12, and 24 months, indicating equivalent long‑term therapeutic efficacy of the two formulations.

**FIGURE 3:**
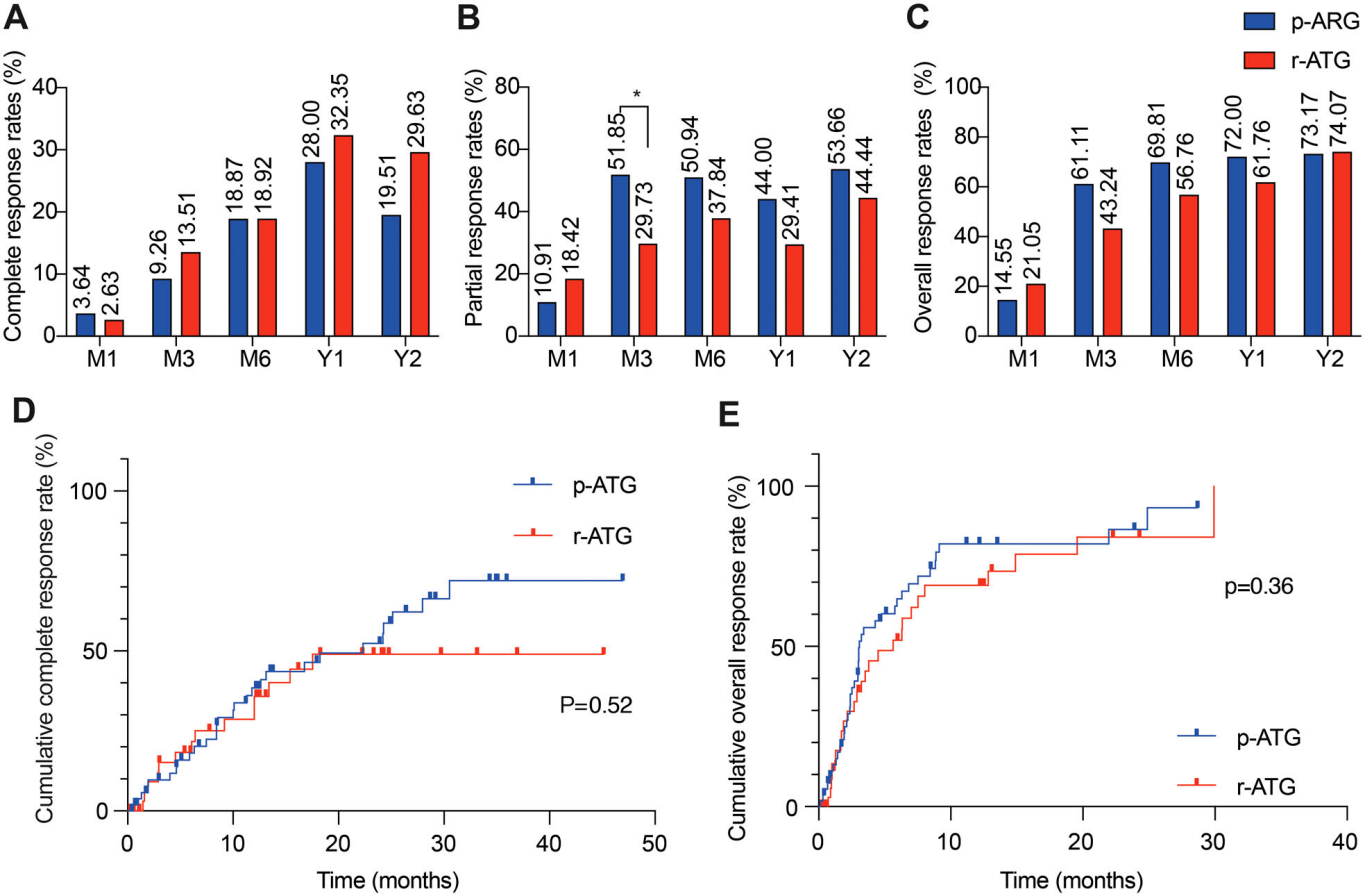
Kinetics of hematologic response of two ATG formulations. (A–C) Hematological responses (CRR, PRR, and ORR) for 1, 3, and 6 months and at 1 and 2 years after IST. (D and E) Cumulative CRR and ORR after IST.

To eliminate potential pharmacodynamic disparities between these two TPO‐RAs, we prespecified subgroup analyses stratified by TPO‐RA agent. Within the eltrombopag subgroup, p‑ATG was associated with superior treatment responses at 3 months (*p* = 0.02) and 1 year (*p* = 0.02) relative to r‑ATG (Figure S2). No statistically significant intergroup differences in treatment efficacy were detected at any of the remaining follow‐up time points. In the herombopag subgroup, overall responses were comparable between the two ATG regimens across all assessed time points.

At 6 months, the cumulative complete response rate (CRR) was 20.23% in the p‐ATG group and 21.70% in the r‐ATG group; at 2 years, these rates were 55.54% and 49.00%, respectively (3D). In contrast, the cumulative response rate was 64.89% with p‐ATG and 51.90% with r‐ATG at 6 months, and 86.48% and 84.01% at 2 years, respectively (Figure 3E).

### 2.5 Survival and Clonal Evolution

The OS time was similar among the groups: 26.40 months (range, 0.40–56.90) in patients treated with p‐ATG and 23.72 months (range, 0.23–48.37) in patients treated with r‐ATG (Figure 4). Of the nine deaths in the p‐ATG group, three resulted from intracranial hemorrhage, five from infection (three from pneumonia and two from septicemia) and one occurred after stem‐cell transplantation. Of the six deaths in the r‐ATG group, two resulted from intracranial hemorrhage, and four resulted from infection (two from pneumonia and two from septicemia). Clonal evolution was assessed in all the patients. One patient in the r‐ATG group progressed to chronic myelomonocytic leukemia (CMML), with a progression‐free survival (PFS) of 20.27 months.

**FIGURE 4:**
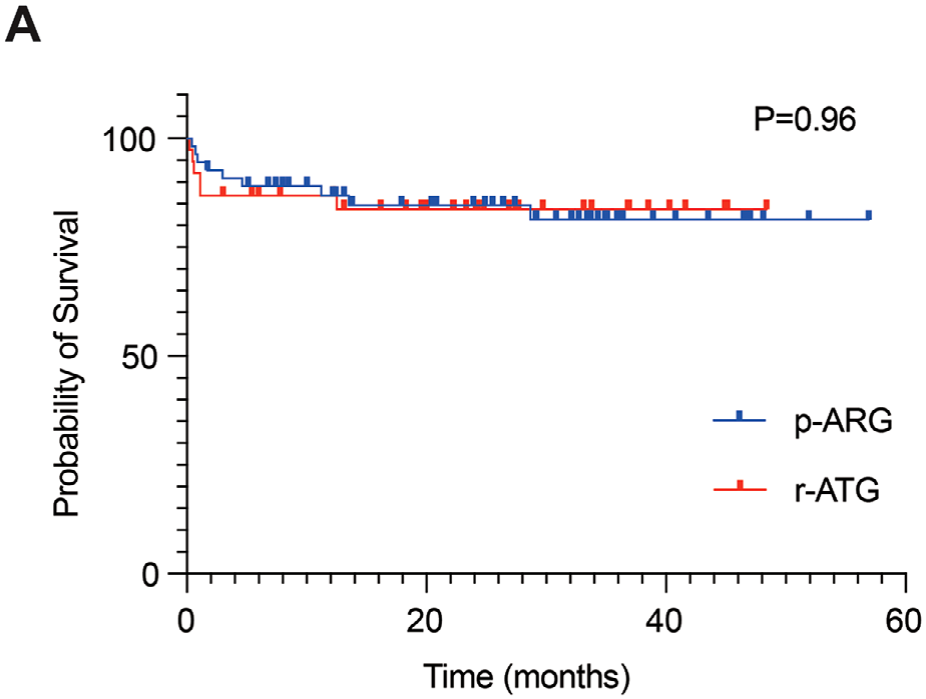
Kaplan–Meier survival curves of overall survival. The log‐rank test showed no statistical difference among the three groups.

### 2.6 Effect of ATGs on Cytokines

We serially evaluated changes in plasma cytokine levels, including IL‐2, IL‐4, IL‐6, IL‐10, TNF‐α, CCL2, IL‐1β, IL‐1RA, and IL‐12p70, using flow cytometric analysis [10]. Notably, only IL‐10 was significantly elevated on Day 2 after ATG administration, likely reflecting a direct pharmacological effect of both p‐ATG and r‐ATG (Figure 5A). However, no significant intergroup differences were observed between the two ATG preparations, except that IL‐10 levels were significantly higher in the r‐ATG group on Day 4 following infusion (Figure 5B and Figure S3).

**FIGURE 5:**
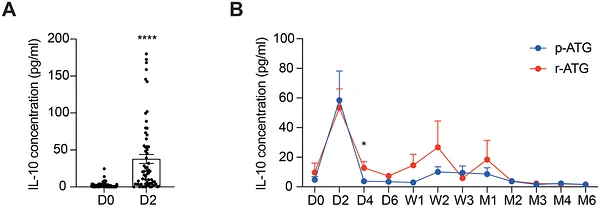
Serum IL‐10 concentration after IST. (A) IL‐10 concentration on Day 0 and 2 in all patients treated with p‐ATG and r‐ATG (*n* = 93). (B) Dynamic changes in serum IL‑10 concentrations from baseline up to 6 months post‑treatment in patients receiving p‑ATG or r‑ATG (*n* = 93).

### 2.7 Infection and Incidence of Serum Sickness

Infection rates between IST were comparable between the two groups (Table 1). Early infection was defined as infection occurring within 1 month after treatment. The incidence of early infection was 40.00% (22/55) in the p‐ATG group and 52.63% (20/38) in the r‐ATG group. Although the rate was numerically higher in the r‐ATG group, there was no statistically significant difference between the two groups.

Serum sickness (SS) usually manifested as fever, arthralgia, malaise, and cutaneous eruptions. In this study, 11 of 55 (20.00%) p‐ATG‐treated patients and three of 38 (7.89%) r‐ATG‐treated patients developed clinical SS, which did not reach statistical significance (*p* = 0.14).

## 3 Discussion

This study identified distinct pharmacokinetic and immunomodulatory profiles between p‐ATG and r‐ATG, while confirming their comparable efficacy and safety in patients with SAA. To our knowledge, this is the first prospective study to compare the in vivo pharmacokinetic profiles of p‐ATG and r‐ATG in patients with SAA. Both formulations reached peak serum concentrations on Day 5 after administration. Still, r‐ATG was cleared significantly more rapidly, which may be attributed to differences in antibody specificity and in vivo metabolic properties. A recent study on h‐ATG demonstrated substantial interpatient variability in exposure, with higher exposure levels associated with earlier hematologic recovery [14]. Notably, peak active ATG concentrations were comparable between the p‐ATG and r‐ATG groups, which partly explains the similar ORRs and OS observed in the two cohorts.

Our prospective study showed the 2‐year ORRs (p‐ATG vs. r‐ATG, 73.17% vs. 74.07%) and overall survival (OS) were similar between the two groups. Notably, the p‐ATG group showed stable absolute neutrophil counts in the first week. In contrast, the r‐ATG group had an early decline in neutrophil counts, which may explain the numerically higher early infection rates observed in this group. The r‐ATG group also exhibited more prolonged lymphopenia and lower CD3^+^ and CD4^+^ T‐cell counts, suggesting a more potent immunosuppressive effect of r‐ATG. These immunoregulatory differences may underlie variations in short‐term immune status but did not translate into long‐term efficacy disparities, which is consistent with some international perspectives that the immunological heterogeneity of ATG formulations does not affect core therapeutic outcomes for SAA [15].

From a clinical perspective, our findings have important practical implications for regions where horse ATG is unavailable. The comparable efficacy and safety profiles of p‐ATG and r‐ATG support their interchangeability in clinical practice, offering greater flexibility in treatment selection when one formulation is unavailable or contraindicated. However, this study has several limitations. Eltrombopag and herombopag were prescribed non‐randomly, with treatment choice decided by individual centers and clinicians. This design inherently causes selection bias and variability in therapeutic practice. The follow‐up duration was insufficient to assess long‐term clonal evolution in detail. In our cohort, no significant difference in clonal evolution was observed between the two groups during the short‐term observation period. However, in a retrospective study, the incidence of MDS/AML transformation was 2.6% and 11.8% among patients treated with p‐ATG and r‐ATG, respectively (*p* = 0.049) [12]. Another long‐term follow‐up study of p‐ATG combined with CsA in patients with SAA found that only 2.0% of patients progressed to MDS after a median follow‐up of 59.6 months [16]. When comparing rabbit and horse ATG, neither head‐to‐head phase III trials nor meta‐analyses have identified a significant difference in the risk of clonal evolution. Therefore, whether the species origin of ATG affects long‐term clonal evolution remains controversial, and extended follow‐up of our cohort is required to address this question. Another limitation of this study is that the trial was registered retrospectively. Also, further large‑sample prospective studies are warranted to explore individualized application strategies for p‑ATG and r‑ATG in specific SAA populations, including elderly patients and those with very severe aplastic anemia, to optimize clinical treatment decisions.

## 4 Conclusion

In conclusion, p‐ATG and r‐ATG exhibit distinct pharmacokinetic profiles and exert differential effects on neutrophil, and lymphocyte counts, as well as the distribution of lymphocyte subsets—differences that may contribute to variations in the intensity of immunosuppression. Importantly, despite these mechanistic disparities, p‐ATG and r‐ATG yield similar ORRs when used as IST for SAA, supporting their interchangeability in clinical practice when specific formulations are unavailable or contraindicated.

## 5 Methods

### 5.1 Patients, Inclusion, and Exclusion Criteria

This study enrolled patients registered at the First Affiliated Hospital of Nanjing Medical University/Jiangsu Province Hospital (Nanjing, China), the Second Affiliated Hospital of Soochow University (Suzhou, China), and was registered at the Chinese Clinical Trial Registry (ChiCTR2200055696). This study protocol was reviewed and approved by the Ethics Committee of the Second Affiliated Hospital of Soochow University (JD‐LK‐2021‐101‐02). Written informed consent was obtained from participants to participate in the study. The study was carried out in accordance with the principles of the Declaration of Helsinki.

The eligibility criteria were as follows: SAA patients who meets the criteria of marrow cellularity < 25% (or 25%–50% with <30% residual hematopoietic cells), plus at least two of the following: absolute neutrophil count (ANC) <0.5 × 10^9^/L, platelets < 20 × 10^9^/L; absolute reticulocyte count (ARC) < 20 × 10^9^/L; VSAA patients were determined as ANC < 0.2 × 10^9^/L; age between 18 and 70 years; red blood cell transfusion dependence and/or platelet transfusion dependence; have not received ATG treatment; ECOG physical status score ≤ 2 points; liver function ALT, AST, TBIL less than two times the upper limit of normal value; and renal function BUN, Cr ≤ 1.25 times the upper limit of normal value. The exclusion criteria were as follows: congenital bone marrow failure diseases, myeloproliferative neoplasms, classic paroxysmal nocturnal hemoglobinuria, and other diseases; not dependent on blood transfusion; patients with other serious diseases, such as serious damage to important organ functions: respiratory failure, heart failure, decompensated liver insufficiency, renal insufficiency, etc.; known to be allergic to ATG or have a history of severe allergy to biological products (including antibiotics).

The primary endpoint was dynamic changes in lymphocyte subsets. Secondary endpoints included pharmacokinetics, neutrophil counts, 6‐month CRR and ORR, SS‐related adverse events, 2‐year ORR, and mortality.

Adverse events were graded according to the National Cancer Institute Common Terminology Criteria for Adverse Events (CTCAE), version 5.0, and tabulated as incidence rates.

### 5.2 Randomization and Treatment Regimens

Randomization was performed centrally using an interactive web‐response system to assign patients in a 1.5:1 ratio to receive P‐ATG or r‐ATG. P‐ATG (Aiteji, Wuhan Zhongsheng Yujin Biomedical) at a dose of 30 mg (/kg×d) or r‐ATG (Thymoglobulin, Genzyme‐Sanofi) at a dose of 3.5 mg (/kg×d) or was administered intravenously for 5 days in combination with CSA. CsA was administered orally at an initial dose of 3–5 mg/kg daily and adjusted as need based on CsA concentrations or side effects, with a target trough concentration of 150–250 µg/L. TPO‐RAs (Eltrombopag or Hetrombopag) were used concurrently with ATG and adjusted according to platelet counts [17]. Eltrombopag was initiated at 25 mg daily and titrated by 25 mg every 3 days to 75 mg daily within 1 week. In comparison, Herquepopa was initiated at 2.5 mg daily and titrated by 2.5 mg every 3 days to 7.5 mg daily within 1 week. Uniform, concomitant supportive care was administered to participants in both treatment arms throughout the study follow‐up period.

### 5.3 ATG Concentration Examination

A sandwich ELISA was used to measure ATG concentrations. Briefly, 96‐well plates were coated with anti‐ATG capture antibody and blocked with 5% skim milk. Serial dilutions of serum samples and ATG standards were added, followed by incubation with HRP‐conjugated secondary antibody. The reaction was developed using a TMB substrate, and the optical density (OD) at 450 nm was measured. Sample ATG concentrations were calculated using a standard curve generated from known ATG standards.

We quantified the percentage of active ATG by using a methodology that integrates flow cytometry and ELISA. Briefly, 100 µL aliquots of PBMCs were added to each well of a 96‐well plate, followed by 10 µL of diluted serum samples or standard solutions. The mixture was incubated at 4°C for 2 h to allow specific binding between active ATG and cell surface antigens. After incubation, cells were centrifuged at 400 g for 5 min; the supernatant was discarded, and 100 µL of FITC‐labeled secondary antibody (goat anti‐porcine IgG‐FITC for p‐ATG samples, goat anti‐rabbit IgG‐FITC for ATG‐F or r‐ATG samples) was added. The plate was incubated at room temperature in the dark for 40–60 min, then cells were centrifuged and washed twice with flow cytometry staining buffer. Finally, cells were resuspended in 200 µL of flow cytometry staining buffer and analyzed using a NovoCyte flow cytometer (Agilent). The mean fluorescence intensity (MFI) was recorded, and a calibration curve was established by linear regression of MFI against the concentration of standard solutions. The concentration of active ATG in serum samples was calculated based on the calibration curve.

### 5.4 Response Evaluation Criteria

The response evaluation criteria were as follows: Complete response (CR) was defined as Hb > 100 g/L, ANC > 1.0 × 10^9^/L, and platelets > 100 × 10^9^/L. Patients with non‐remission (NR) still satisfied the SAA standard. Partial response (PR) is the condition between CR and NR. OS is defined as the achievement of either CR or PR [6, 18].

### 5.5 Statistical Analysis

The planned sample size of 98 patients yielded 80% statistical power at a two‐sided α of 0.05 to detect a difference in Day 3 lymphocyte proportions between the r‐ATG group (15%, 0.15) and the p‐ATG group (40%, 0.40). The data were analyzed using SPSS Statistics (version 26.0) and GraphPad Prism (Version 10.3.0). Continuous variables are presented as mean ± standard deviation or median (interquartile range), while categorical variables are expressed as frequencies and percentages. For continuous variables, normality of distribution was first assessed. Data conforming to a normal distribution were compared between the two groups using the unpaired *t*‐test, whereas the Mann–Whitney *U* test was applied for non‐normally distributed data. Hedges’ g (standardized mean difference [SMD]) was calculated to quantify the magnitude of intergroup baseline imbalance, and an absolute SMD < 0.1 was defined as indicative of adequate baseline balance. All unbalanced covariates were adjusted in multivariate models for subsequent efficacy analyses. The chi‐square test or Fisher's exact test was used for the comparison of categorical variables. OS and the corresponding 95% confidence intervals (CIs) were estimated using the Kaplan–Meier method. Potential risk factors were identified using binary logistic regression analysis. A two‐sided *p* value < 0.05 was considered statistically significant.

## Author Contributions

G.H. and Y.S. conceived the study. Yawen Zhang, Yingying Zhang, and Y.G. collected the clinical and laboratory data. Yawen Zhang assisted with data interpretation. Yawen Zhang wrote the manuscript with the help of all the authors. All authors have reviewed and approved the final submitted manuscript and agreed to its publication.

## Funding

This study was supported by the National Natural Science Foundation of China (82400138 and 81900109) and Natural Science Foundation of Jiangsu Province (BK20230734).

## Ethics Statement

This study protocol was retrospectively registered at the Chinese Clinical Trial Registry (ChiCTR2200055696) and approved by the Ethics Committee of the Second Affiliated Hospital of Soochow University (JD‐LK‐2021‐101‐02). Written informed consent was obtained from participants to participate in the study. The study was carried out in accordance with the principles of the Declaration of Helsinki.

## Conflicts of Interest

The authors declare no conflicts of interest.

## Supporting information




**Figure S1** Flow diagram of the clinical trial of two ATG formulations.
**Figure S2** Kinetics of hematologic response of two ATG formulations with different TPO‐RAs: (A) Eltrombopag group (p‐ATG, *n* = 36; r‐ATG, n = 22); (B) Hetrombopag group (p‐ATG, *n* = 19; r‐ATG, *n* = 16).
**Figure S3** Dynamic changes in serum cytokine concentrations from baseline up to 6 months post‑treatment in patients receiving p‑ATG or r‑ATG.

## Data Availability

The trial protocol and the datasets used and analyzed during the current study are available from the corresponding author on reasonable request.
