# Supplementary material for: Safety, Pharmacokinetics, and Efficacy Study of Porcine and Rabbit Antithymocyte Globulin in the Treatment of Patients With Severe Aplastic Anemia: A Multicenter, Prospective, Randomized, Open‐Label Clinical Trial
Source: MedComm (2020). 2026 Oct 17;7(11):e71084. doi: 10.1002/mco2.71084 (PMC13676421; doi:10.1002/mco2.71084)
Supplement: Supplementary file 1 [file MCO2-7-e71084-s001.docx]

**Safety, Pharmacokinetics and Efficacy study of Porcine and Rabbit Antithymocyte Globulin in the treatment of patients with Severe Aplastic Anemia: A Multicenter, Prospective, Randomized, Open-Label Clinical Trial**

Yawen Zhang^1#^, Yingying Zhang^2#^, Yuemin Gong^1^, Yu Sun^2*^ and Guangsheng He^1*^

^1^Department of Hematology, Jiangsu Province Engineering Research Center of Cell and Gene Therapy for hematologic and lymphoid Diseases, The First Affiliated Hospital with Nanjing Medical University, Jiangsu Province Hospital, Nanjing, China

^2^Department of Hematology, The Second Affiliated Hospital of Soochow University, Suzhou, China

Yawen Zhang and Yingying Zhang contributed equally to this work.

Correspondence: Guangsheng He, Email: heguangsheng1972@sina.com;

Yu Sun, Email: sunyu2084@suda.edu.cn;

Key words: immunosuppressive therapy; severe aplastic anemia, clinical trial, porcine antithymocyte globulin; rabbit antithymocyte globulin

**Figure S1** Flow diagram of the clinical trial of two ATG formulations

**Figure S2** Kinetics of hematologic response of two ATG formulations with different TPO-RAs (A) Eltrombopag group (p-ATG, n=36; r-ATG, n=22); (B)Hetrombopag group (p-ATG, n=19; r-ATG, n=16)

**Figure S3** Dynamic changes in serum cytokine concentrations from baseline up to 6 months post‑treatment in patients receiving p‑ATG or r‑ATG.
